# Infantile epileptic spasms syndrome as an initial presentation in infantile choroid plexus papilloma: A case report

**DOI:** 10.3389/fped.2022.1035621

**Published:** 2022-11-16

**Authors:** Faliang Zhou, Yu Li, Lixue Shen, Hongxin Yao, Xinlin Hou

**Affiliations:** ^1^ Department of Pediatrics, Peking University First Hospital, Beijing, China; ^2^ Department of Pediatric Surgery, Peking University First Hospital, Beijing, China

**Keywords:** choroid plexus papilloma, ventriculomegaly, west syndrome, infantile spasms (IS), infantile epileptic spasms syndrome

## Abstract

We present an interesting report of a 5-month-old infant with epileptic spasms and developmental delay who presented with non-isolated ventriculomegaly in utero and whose brain magnetic resonance imaging revealed right ventricular choroid plexus papilloma (CPP). The epileptic spasms persisted even with the use of antiepileptic therapies but was apparently cured after the removal of a CPP.

## Introduction

Choroid plexus papilloma (CPP) develops as an intraventricular neoplasm arising from the epithelium of the choroid plexus and affects children and infants disproportionately with an incidence of 4% in all pediatric brain tumors (1, 2). CPP is commonly found in the lateral ventricles with a rate of 43%–65%, followed by the fourth and the third ventricle, and less in other regions (3, 4). Hydrocephalus, macrocephaly, enlarged fontanelles, and symptoms of intracranial hypertension are noted in nearly all clinical cases with CPP (5, 6). However, infantile epileptic spasms syndrome as the initial clinical finding in such patients is rare, and only a few cases have been reported in the literature (7). However, an infantile CPP without hydrocephalus presentation combined with infantile epileptic spasms syndrome was not reported in the publication. In the present study, we describe a patient aged 5 months who presented with epileptic spasms initially and found CPP with non-isolated ventriculomegaly in utero.

## Case presentation

**Figure 1:**
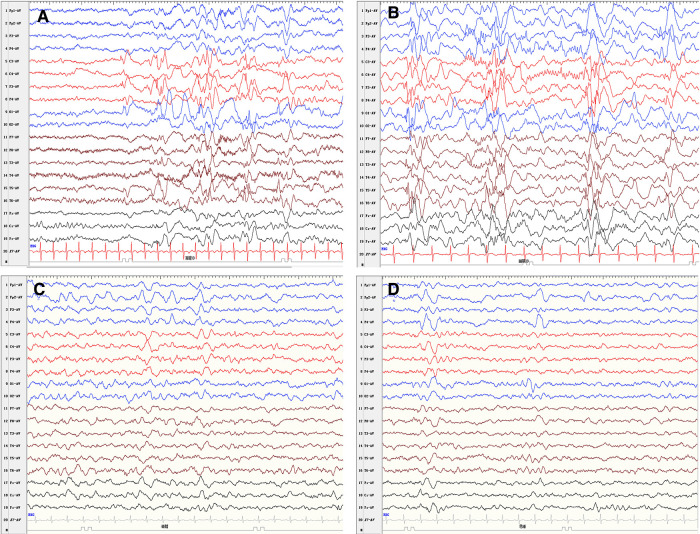
Electroencephalogram findings. (**A**) Low amplitude fast waves superimposed over diffused slow wave activity. (**B**) Hypsarrhythmia. EEG findings improved dramatically at 2 months after the surgery (**C, D**).

A 5-month-old female patient was admitted to the hospital due to epileptic spasms. Her growth and development within 3 months were normal. She could not roll over and raise her head steadily while lying on her back, which meets the diagnostic criteria of development retrogression and delay at 5 months of age. Hypsarrhythmia was observed on the interictal electroencephalogram (EEG). Based on this EEG pattern (Figure 1), the patient was diagnosed with infantile epileptic spasms syndrome. Epileptic spasms were manifested as rhythmic fast waves in the left temporal and occipital region. Levetiracetam (LEV) and valproic acid (VPA) failed to control the seizures. Complete blood count, liver and kidney function, electrolyte, blood coagulation test, metabolic screening, and whole-exome sequencing were normal. A comprehensive examination of the eyes was unremarkable.

**Figure 2:**
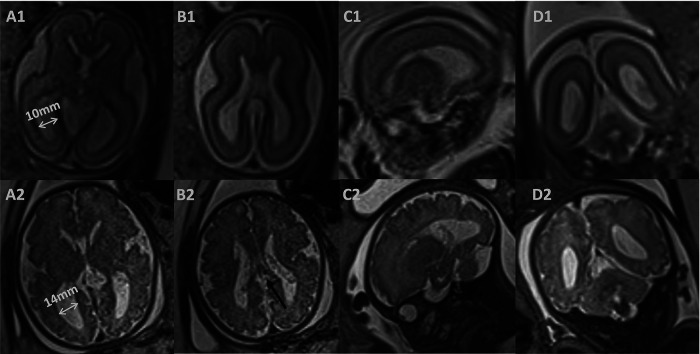
Prenatal magnetic resonance images. (1) T2-weighted image at 25 weeks of gestation. (2) T2-weighted image at 35 weeks of gestation. Axial (**A, B**) and coronal (**D**) images showed symmetrically dilated lateral ventricles, and corpus callosum agenesis (black arrow); sagittal (**C**) images showed abnormal morphology of the right ventricle.

She was delivered naturally at 41 weeks of gestation with a weight of 3470 g and an Apgar score of 10 at both 1 and 5 min. The physical and neurological examinations were normal at birth. Fetal ventriculomegaly was detected at 22 weeks of gestation. An amniocentesis showed a normal karyotype and chromosomal microarray analysis. The TORCH screening test showed a negative result. A prenatal ultrasound had been performed weekly to monitor the fetal ventriculomegaly. The lateral ventricle atrium width was shown to progressively enlarge from 10 mm at 22 weeks to 14 mm at 35 weeks. Furthermore, fetal brain magnetic resonance imaging (MRI) was performed at 25 and 35 weeks, describing symmetrically dilated lateral ventricles, corpus callosum agenesis, and abnormal morphology of the right ventricle (Figure 2). The brain parenchyma volume and morphology were normal. The choroid plexus presented a normal signal intensity on all sequences and there were no signs of intraventricular hemorrhage or plexus cysts. She had an unremarkable familial and personal history.

**Figure 3:**
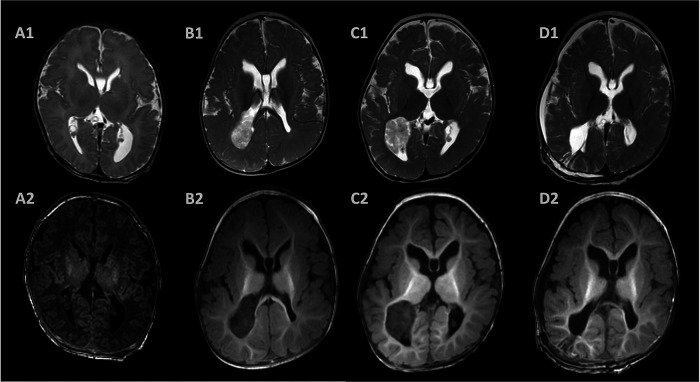
Postnatal magnetic resonance images. The brain MRI showed dilated ventricles, corpus callosum agenesis, and subependymal pseudocyst 3 days after birth (**A**). A large mass located in the posterior horn of the right ventricle with T2 heterogeneously hyperintense and T1 hypointense at 6 months (**B**) and 10 months (**C**) after birth with progression. T2- and T1-weighted images showed a Normal appearing choroid plexus and porencephalic cavity 2 weeks after surgery (**D**).

Due to the evaluation of the fetal ventriculomegaly, the brain MRI 3 days after birth showed a dilated left ventricle, corpus callosum agenesis, and subependymal pseudocyst. In addition, the cranial ultrasound provided the same results at the age of 3 days, 1 month, and 3 months. We performed a brain MRI scan after the diagnosis of infantile epileptic spasms syndrome. However, a large mass in the posterior horn of the right lateral ventricle with T2 heterogeneously hyperintense and T1 hypointense was observed in the brain MRI scan and considered to be a CPP (Figure 3). A large mass was removed through a right parietal craniotomy and the resected tissue was received in fragments aggregating to 2.5 cm × 2.0 cm × 2.0 cm. Histopathologic examination and immunoperoxidase staining showed the mass was a CPP.

**Figure 4:**
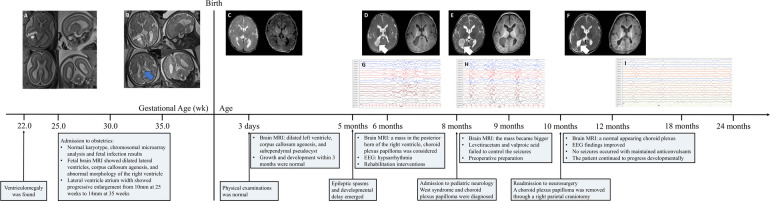
Clinical course of the patient and identification of a choroid plexus papilloma in the panel, the patient's mother was admitted to our institution for prenatal counseling due to fetal ventriculomegaly at 22 weeks of gestation. Amniocentesis showed a Normal karyotype and chromosomal microarray analysis. Fetal infection results were within Normal limits. fetal brain magnetic resonance imaging was performed at 25 and 35 weeks, describing symmetrically dilated lateral ventricles, progressive enlargement (from 10 mm at 22 weeks to 14 mm at 35 weeks), corpus callosum agenesis (blue arrow), and abnormal morphology of the right ventricle (subpanels **A, B**). A diagnosis of non-isolated ventriculomegaly was made and a female was delivered vaginally with Normal physical and neurological examinations. Brain MRI 3 days after birth showed dilated left ventricle, corpus callosum agenesis, and subependymal pseudocyst (subpanel **C**). At 5 months of age, epileptic spasms and developmental delay emerged, and hypsarrhythmia was evident on electroencephalogram (subpanels **G and H**). Levetiracetam and valproic acid failed to control the seizures. Routine laboratory tests and whole-exome sequencing were unremarkable. A large mass located in the posterior horn of the right ventricle with T2 heterogeneously hyperintense and T1 hypointense at 6 months (subpanel **D**) and 10 months (subpanel **E**) after birth with progression. A large mass was removed through a right parietal craniotomy and the histopathologic examination showed the mass was a choroid plexus papilloma. T2- and T1-weighted images showed a Normal appearing choroid plexus and porencephalic cavity 2 weeks after surgery (subpanel **F**). The child was maintained postoperatively on anticonvulsants and no seizures occurred. Throughout follow-up, he showed continuing developmental improvement. EEG findings improved dramatically at 2 months after the surgery (subpanel **I**).

The patient was followed up with a brain MRI scan 2 weeks after surgery, which showed a normal-appearing choroid plexus. The LEV and VPA were withdrawn gradually 6 months after the operation and were seizure-free. Throughout the follow-up, she has continued to progress developmentally. An improvement in fine and gross motor skills was observed by the parents, although both mental developmental index and psychomotor developmental index scores in the Bayley scales of infant development were lower than 70 at the age of 15 months. An EEG performed 2 months after surgery showed a significant improvement (Figure 1). The detailed clinical course of the patient is shown in Figure 4.

## Discussion

CPP is a rare intracranial tumor and is classified as grade I by the World Health Organization classification of tumors. In the pediatric age group, half occur in the first year of life (4). At any time of diagnosis, hydrocephalus is noted in almost all cases due to much cerebrospinal fluid (CSF) produced by the tumor and the blocked CSF circulation pathway (5). The common signs and symptoms are unusually large hands, bulging or tense fontanelle, poor feeding, irritability, vomiting, sleepiness, and so on. Computed tomography scan reveals iso- or high-density masses, associated with calcification or hemorrhage occasionally; MRI shows a slightly low signal intensity on T1-weighted imaging and a high signal intensity on T2-weighted imaging (8).

Hydrocephalus and symptoms of intracranial hypertension are the main clinical findings in almost all patients with CPP. The relation between infantile epileptic spasms syndrome and CPP was less reported in previous literature. Branch et al. (7) described a 7-month-old infant who had infantile epileptic spasms syndrome with hydrocephalus and was finally diagnosed with CPP; removal of a CPP of the left lateral ventricle was followed by a clinical recovery free of seizures using anticonvulsant therapy. Our case demonstrates similar clinical features compared to the prior reports but differs from the patient with the markedly dilated left ventricle with a mild shift of the midline contents in the literature.

Adrenocorticotropic hormone therapy and vigabatrin are the combination treatment suggested to be superior for patients with infantile epileptic spasms syndrome (9). Specifically, vigabatrin is the preferred treatment for patients with tuberous sclerosis complex. In addition, hormonal treatment is the preferred initial method for most other patients, especially in cryptogenic cases (10). The case described by Branch et al. (7) showed that the patient with epileptic spasms caused by CPP could be cured by removing the tumor, but the limited improvement was produced by adrenocorticotropic hormone therapy (80 units per day) and antiepileptic drugs. In this case, LEV and VPA were taken as the first treatment because of the accessibility of the drug compared with hormone therapy and vigabatrin at the time. Due to the rapid progression of the tumor, we decided to carry out surgical treatment first, and then determine whether further antiepileptic therapies were needed depending on the therapeutic efficacy of the seizure. However, the child was seizure-free and this was maintained postoperatively with LEV and VPA reduced gradually. The course of these patients illustrates that a thorough diagnostic evaluation should be undertaken of all cases of infantile spasms for pursuing curative treatments.

Aicardi syndrome is a differential diagnosis in this case because of the epileptic spasms and corpus callosum agenesis. CPP has also been described in Aicardi syndrome (11). A pathogenic gene for Aicardi syndrome has not yet been identified, but several observations support a hypothesis that Aicardi syndrome is caused by de novo pathogenic variants in a gene on the X chromosome that is subject to X chromosome inactivation (12). However, in our case, the patient was female, without central chorioretinal lacunae, and whole-exome sequencing of our patient was unremarkable. Therefore, Aicardi syndrome should be ruled out.

Approximately 5% of perinatal brain tumors and 10% of all brain tumors in infants are of choroid plexus etiology (5). In these cases, ultrasound demonstrated large, echogenic choroid plexus with associated ventriculomegaly (13, 14). However, no publications reported an infantile CPP combined with ventriculomegaly in utero. In the case reported here, an infantile CPP was diagnosed at the age of 5 months with epileptic spasms, and the ventriculomegaly was found in the fetal MRI scan combined with corpus callosum agenesis and abnormal morphology of the right ventricle, but without a mass in the lateral ventricle. The condition needs to be considered because fetal ventriculomegaly occurs due to too much CSF produced by the tumor.

In conclusion, it is extremely rare that infantile epileptic spasms syndrome is the initial clinical finding in patients with CPP without hydrocephalus. Imaging methods are effective for a differential diagnosis. Surgery is the mainstay of treatment for seizure control. In addition, the fetus with progressive, non-isolated ventriculomegaly in utero needs follow-up closely after birth.

## Data Availability

The original contributions presented in the study are included in the article/Supplementary Material, further inquiries can be directed to the corresponding author/s.
